# Angular momentum transfer from photon polarization to an electron spin in a gate-defined quantum dot

**DOI:** 10.1038/s41467-019-10939-x

**Published:** 2019-07-16

**Authors:** Takafumi Fujita, Kazuhiro Morimoto, Haruki Kiyama, Giles Allison, Marcus Larsson, Arne Ludwig, Sascha R. Valentin, Andreas D. Wieck, Akira Oiwa, Seigo Tarucha

**Affiliations:** 1 Department of Applied Physics, The University of Tokyo, 7-3-1 Hongo, Bunkyo-ku, Tokyo 113-8656 Japan; 2 The Institute of Scientific and Industrial Research, Osaka University, 8-1 Mihogaoka, Ibaraki, Osaka 567-0047 Japan; 3 Center for Emergent Matter Science (CEMS), RIKEN, 2-1 Hirosawa, Wako-shi, Saitama, 351-0198 Japan; 4 Lehrstuhl für Angewandte Festkörperphysik, Ruhr-Universität Bochum, Universitätsstraße 150, Gebäude NB, D-44780 Bochum, Germany; 5 Center for Spintronics Research Network (CSRN), Graduate School of Engineering Science, Osaka University, Osaka, 565-0871 Japan; 6 Quantum Information and Quantum Biology Division, Institute for Open and Transdisciplinary Research Initiatives, Osaka University, Osaka, 565-0871 Japan

**Keywords:** Nanoscience and technology, Electronic devices, Optics and photonics, Physics

## Abstract

Gate-defined quantum dots (QDs) are such a highly-tunable quantum system in which single spins can be electrically coupled, manipulated, and measured. However, the spins in gate-defined QDs are lacking its interface to free-space photons. Here, we verify that a circularly-polarized single photon can excite a single electron spin via the transfer of angular momentum, measured using Pauli spin blockade (PSB) in a double QD. We monitor the inter-dot charge tunneling which only occur when the photo-electron spin in one QD is anti-parallel to the electron spin in the other. This allows us to detect single photo-electrons in the spin-up/down basis using PSB. The photon polarization dependence of the excited spin state was finally confirmed for the heavy-hole exciton excitation. The angular momentum transfer observed here is a fundamental step providing a route to instant injection of spins, distributing single spin information, and possibly towards extending quantum communication.

## Abstract (web summary)

Gate-defined quantum dots offer a way to engineer electrically controllable quantum systems with potential for information processing. Here, the authors transfer angular momentum from the polarization of a single photon to the spin of a single electron in a gate-defined double quantum dot.

## Introduction

Semiconductor quantum dots (QDs) is an appropriate platform for hosting photons and spins and therefore can provide a quantum interface for converting information media between photon polarization and spin orientation^1–9^. This process follows the selection rule in optical transitions to create an electron–hole pair or an exciton from a photon polarization while preserving angular momenta, and has long been studied for quantum wells by optical means^10–12^. However, to assess a photon-to-spin interface, it is necessary to create the single electron spins with various photon polarization and electrically measure the spin orientation before it relaxes. Laterally gated QDs in a quantum well may be appropriate for these purposes because the orbital and spin angular momenta are well-defined by the strong vertical confinement and suitable for the transition with angular momentum selection rule, while the soft lateral confinement will least affect the selection rule for the optical excitation. Investigating the singly excited charged spin electrically is essential for understanding the selection rule in the optical excitation, robustness of the photo-generated electron spin, and for applications in nano-scaled spin-based information processing^13,14^, but a challenging task.

Single electrons are optically accessible in gate-defined QDs because the electronic potential acts as a trapping potential for the electrons, while it is repulsive for the holes upon single electron–hole pair excitation in the QD area^15,16^. Then single photo-electrons can be generated from the individual heavy-hole (HH) and light-hole (LH) excitons^17,18^, and detected using a nearby charge sensor because the photo-electron trapping results in a charge addition for the dot. The photo-electron spin orientation can be judged using the spin-dependent tunneling from the dot to the lead whose Fermi energy is aligned between the Zeeman sub-levels in the dot^19^. However, the Fermi energy has been known to fluctuate due to the photon absorption in the leads outside the dot which has hindered the detection of the spin state^15–17^.

In this work, we measure the single excited spin of a photo-electron in real-time using Pauli spin blockade (PSB) spin readout. We adjust a double QD (DQD) to the inter-dot tunneling resonance condition, which is sensitive to the energy misalignment between the dots. The photo-excited charge moving back-and-forth between the dots enables us to keep track of the suitable condition for a PSB spin readout even when the Fermi energies of the leads connected to either the DQD or charge sensor fluctuate. Finally using this reliable condition, we verify the principle selection rule from a single circularly polarized photon to the excited single electron spin state at the HH exciton by rotating the incident polarization.

## Results

### Optical selection rules of GaAs HH states

**Fig. 1:**
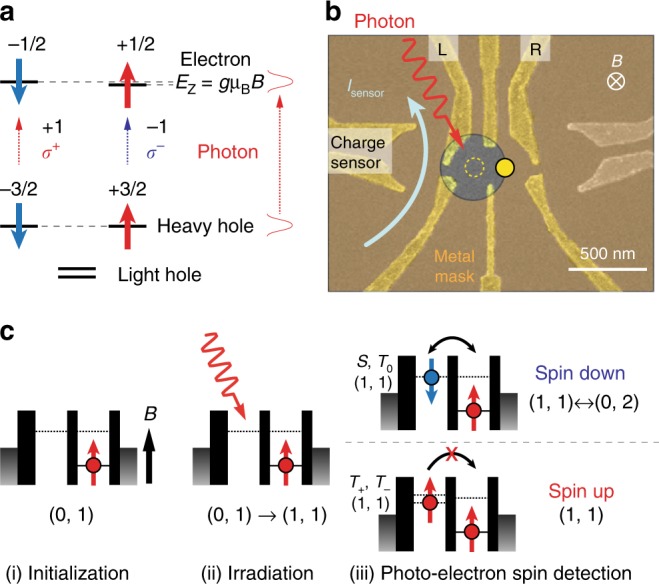
Photo-electron spin detection scheme. **a** Angular momentum conservation in the heavy-hole band excitation. The numbers are the angular momenta of the respective particles in units of *ħ*. **b** Scanning-electron-micrograph picture of the double quantum dot (DQD) gate pattern nominally identical to the measured sample. We mainly use the charge sensor quantum dot (QD) formed on the left. The actual sample has a metal mask placed on an insulator film. Photons are irradiated onto the left dot through an aperture in the metal mask in transparent yellow. **c** Sequence of the single photo-electron spin measurement: (i) Initialization to (0,↑) by waiting long enough at the (0,1) charge state, (ii) exciting an electron–hole pair. We post-select the events where a single electron is captured in the left QD, and (iii) single photo-electron-spin measurement. Spin-down and up are distinguished by observing the charge change due to the (1,1)-(0,2) transition. The DQD is initialized back to (0,↑) by waiting for the excess electron to escape from the DQD

Figure 1a depicts the conversion rule that holds for the QW device. We here focus on the conservation of angular momentum in the electron–hole pair photo-excitation. We photo-excite the lowest energy HH state for simplicity and also to achieve the high efficiency of excitation. The numbers in the figure represent the angular momenta that are defined as positive (negative) for parallel (anti-parallel) to the light propagation direction. The angular momentum is conserved in the optical transition such that a photon with angular momentum −1 (+1) for *σ*^−^ (*σ*^+^) polarization, creates an electron with spin +1/2 (−1/2) and a HH with projection of total angular momentum −3/2 (+3/2), all expressed in units of *ħ*. In other words, $|\sigma ^ - > _{{\mathrm{ph}}} \rightarrow |\!+\! 1/2 > _{\mathrm{e}} \otimes |\! -\! 3/2 > _{{\mathrm{HH}}},|\sigma ^ + > _{{\mathrm{ph}}} \rightarrow | \!-\! 1/2 > _{\mathrm{e}} \otimes | \!+\! 3/2 > _{{\mathrm{HH}}}$.

### Single-spin detection using PSB

For measuring the photo-generated single electron spin, we utilize the mechanism of PSB while monitoring the electron move inside the DQD (Fig. 1b, c). First we place a single electron on the right dot and then the electron spin is initialized to the up-spin state under a sufficiently large magnetic field. This sets the read-out basis vectors of up/down spin for the photo-electron. The left dot is emptied for the photo-electron to be trapped. After a successful trapping, the DQD forms (1,1), where (*n, m*) denotes the set of electron numbers in the left and right QD, respectively. The (1,1) and (0,2) state simultaneously align upon the single photo-electron trapping, energetically allowing transition between the two states^20^. This transition is repeated when the two spins are anti-parallel, but blocked when they are parallel because of the PSB mechanism (explained below). Considering again the readout basis, this difference allows us to measure the spin orientation of the photo-electron as up or down, respectively. Note that the hole is also photo-created in the dot but quickly escapes to the reservoir in the QW^16^.

PSB prohibits charge tunneling between the dots when the tunneling requires spin flip. Starting from the tunnel-coupled two-electron state of (1,1), the ground state is either a spin-singlet state, *S* = (|↑>|↓>−|↓>|↑>)/√2, or a spin-triplet state of *T*_0_ = (|↑>|↓> + |↓>|↑>)/√2, *T*_+_ = |↑>|↑>, and *T*_−_ = |↓>|↓>. Due to PSB, only *S*(1,1) can make a transition to the (0,2) state that is *S*(0,2). In the absence of an external magnetic field all these spin states can be mixed up because of the fluctuating Overhauser field by a few mT in the abundant nuclear spin bath^21^. Therefore, for any spin states of (1,1), the electron inter-dot tunneling time is more or less the same. In the presence of an external magnetic field well exceeding a few mT, the *T*_+_ and *T*_−_ states are separated by Zeeman energy, while the *S* and *T*_0_ states of (1,1) both having the zero magnetic moment are still degenerate. In such a condition the inter-dot electron tunneling is spin blocked for the parallel spin-triplet states, whereas not for the non-magnetic anti-parallel spin states. This difference can be distinguished by charge sensing within the spin relaxation time.

**Fig. 2:**
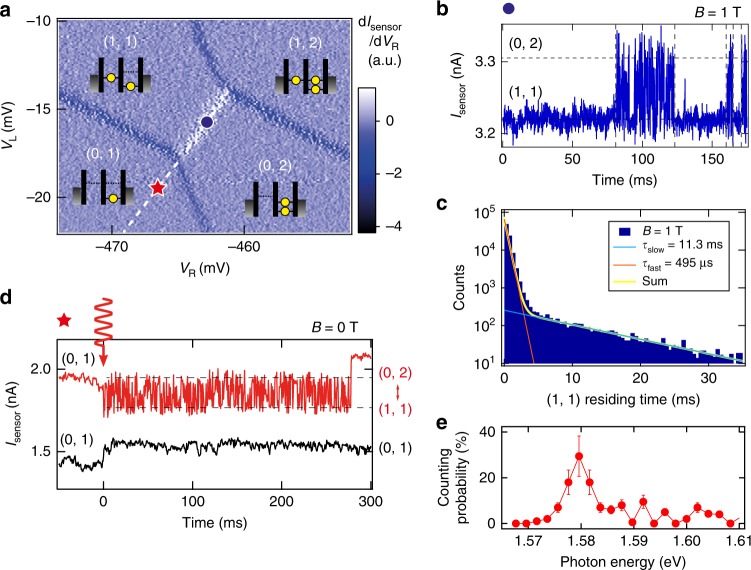
Real-time detection of Pauli spin blockade and single-shot photo-electron trapping. **a** Charge stability diagram measured around the (1,1)-(0,2) transition line in white and the (0,1) region. **b** Example trace of a real-time charge sensor current *I*_sensor_ measured at point ● in the stability diagram with *B* = 1 T. The trace starts from the *I*_sensor_ blocked at a lower value indicating (1,1) and frequently changes between the low and high values indicating the repeated (1,1)-(0,2) transitions. The two regions indicate the parallel and anti-parallel spin configurations, respectively. **c** Histogram of the number of events of finding the (1,1) blocked state vs. the state residing time derived from the *I*_sensor_ trace measured continuously for minutes. The histogram fits a double-exponential curve with two time constants, *τ*_slow_ and *τ*_fast_. These values are used to optimize threshold time for the single-shot spin measurements (see text). **d** Photon irradiation results at *B* = 0 T measured at point ★ in the stability diagram. *I*_sensor_ in red oscillates due to the repeated (1,1)-(0,2) transitions. The two-electron charge dynamics is observed until one of the two electrons escapes from the DQD. *I*_sensor_ in black stays at the low level showing no photo-electron trapping. A small offset of *I*_sensor_ observed for *t* > 0 ms is due to the small photoconductivity of the charge sensor. **e** Energy spectrum of the photo-electron trapping probability. The laser power is tuned such that ~20 photons reach the QD area per shot for this figure. The heavy-hole peak is found at 1.579 eV, and the light hole peak is expected to be at 1.602 eV from simulation for the 7.3 nm-width quantum well (QW) but not well resolved. Error bars are standard deviations expected from the binomially distributed single-shot results of photo-electron detection

We carefully adjust the gate voltages of the DQD device to make appropriate the dot potentials, dot–lead and dot–dot tunnel couplings, and Zeeman energies for the spin readout measurement^19,22^. Then the measured charge stability diagram in the vicinity of the inter-dot transition line between (1,1) and (0,2) is shown in Fig. 2a. We set the gate bias to point ● on the border of (1,1) and (0,2) to monitor the electron inter-dot tunneling events. Here we set the inter-dot tunneling times above milliseconds longer than the integration time of the charge sensor to observe the inter-dot electron move in real time. Figure 2b shows an example of the time-dependent charge sensor current *I*_sensor_ for *B* = 1 T. We observe two conditional regions: the first shows a number of consecutive *I*_sensor_ spikes indicating repeated transitions between the spin-anti-parallel states of (1,1) and (0,2), while the second shows *I*_sensor_ staying at the low-level indicating the spin-parallel (1,1) states blocked due to PSB. We measure the (1,1) residing time and count the number of events to construct a histogram of the (1,1) residing time. We can fit the histogram to a double-exponential curve with two time constants (Fig. 2c): blockade lifetime *τ*_slow_ of parallel spins and spin-preserved tunneling time *τ*_fast_ of anti-parallel spins. We use the ratio of *τ*_slow_ and *τ*_fast_ to calculate a suitable threshold time for judging the spin orientation from the charge sensing data so as to minimize the spin measurement error for arbitrary two-electron states of (1,1) (see Supplementary Note 2 for details). In addition, the read-out fidelity can be increased by making the magnetic field larger than that arising from the fluctuating hyperfine interaction and spin–orbit interaction^19,22^.

### Single photo-electron trapping and its spin detection

In contrast, when trapping a photo-electron, an electron is added to the dot whose chemical potential is located above the Fermi level of the reservoir. Such a non-equilibrium state does not appear in the stability diagram in Fig. 2a, but it is still possible to approximate the gate voltages needed to make alignment of (1,1) and (0,2) upon photo-excitation, for instance those at point ★, which is inside the (0,1) region and along the dashed line. Figure 2d shows typical *I*_sensor_ responses when a laser pulse is irradiated at *t* = 0 with *B* = 0 T, showing a suitable single photo-electron trapping in the upper trace and no photo-electron trapping in the lower trace. We reconfirm the (1,1)-(0,2) degenerate condition by observing the back-and-forth inter-dot electron tunneling events subsequent to the photon irradiation. The escape time of the excess electron is about a few 100 ms restricted by the thicker outer barrier. We note that after retaining the initial (0,1) state no electron enters the DQD from the reservoir, assuring the charge change observed in *I*_sensor_ is only due the photo-electron trapping. The small feature in *I*_sensor_ upon irradiation is attributed to the effect of persistent photo-conductivity, where we see an increase step of *I*_sensor_ from the low-level followed by a gradual decrease. The persistent component accumulates in every shot but is partially compensated by weakly illuminating a longer wavelength light (here a 1550 nm wavelength light from a light-emitting diode) to de-excite the impurities in the heterostructure^23^. Additionally, trapping of multiple photo-electrons can also be detected with a very small probability, and therefore we disregard such events^20^. Figure 2e shows the counting probability of detecting the photo-electron trapping events as a function of the incident photon energy. The spectrum indicates the peak position of the HH exciton at 1.579 eV. We set the photon energy to this peak in the experiment of spin readout of a single photo-electron. The LH exciton is expected to be at around 1.602 eV from simulation and additional measurements^18^, but not well resolved.

### Angular momentum transfer

**Fig. 3:**
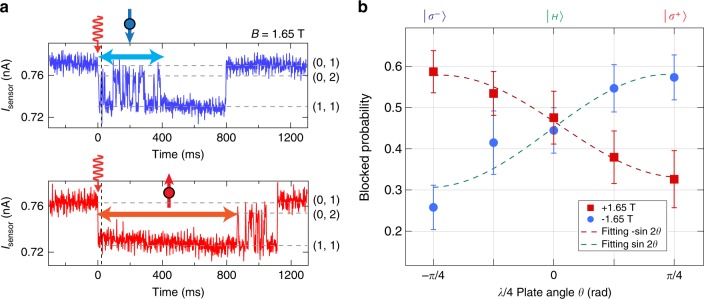
Single photo-electron spin detection and angular momentum transfer. **a** Two typical *I*_sensor_ traces of photo-electron trapping, indicating down-spin trapping in the upper panel, and up-spin trapping in the lower panel, respectively. **b** Polarization and field polarity dependence of the blocked probability, reflecting the angular momentum transfer from a single photon to an electron spin. A sinusoidal dependence is observed against the rotation angle *θ* of polarization from −*π*/4 to *π*/4. By reversing the external magnetic field or reversing the polarity of the spin detection axis, we observe a flipped curve. This means that a single photon polarization creates a single fixed electron spin in the dot, following the conversion rule depicted in Fig. 1a. Each data point is derived from the single-shot measurements repeated more than 1000 times. The photo-electron trapping rate averaged over all measurements is 3%. The error bars ~5% are the standard deviation of the binomial distribution for each measurement point. From the fitting to the sinusoidal curve an amplitude of 25 ± 2% (27 ± 5%) and an offset of 45 ± 1% (44 ± 2%) for *B* = 1.65 T (−1.65 T) are obtained. We used these parameters for fidelity calculation

We finally perform the photo-electron trapping experiment to study the angular momentum transfer from a circularly polarized photon to an electron spin. The magnetic field *B* is increased to 1.65 T to facilitate the initialization of (0,1) having a spin-up electron (i.e. (0,↑)) by setting Zeeman energy larger than the electron temperature. Figure 3a shows the anti-parallel (parallel) spin state detection upon irradiating linearly polarized photons, in the upper (lower) panel of *I*_sensor_. The photo-generation of the (1,1) anti-parallel spin state is discerned by observing the repeated events of inter-dot electron tunneling while keeping the spin orientation, and then relaxing to the (1,1) parallel spin state as a spin-blocked state, and finally restoring the initial (0,1) state by the escape of one electron to the lead. In contrast, the lower panel of *I*_sensor_ shows the opposite behavior, indicating the photo-generation of the parallel spin state *T*_+_. Given that (0,↑) is initially formed, by the photo-excitation the upper (lower) panel indicates the photo-generation of a spin-down (spin-up) electron. *B* in a perpendicular configuration cannot be very large, otherwise the orbital excited (0,2) spin-triplet states become so close to *S*(0,2) in energy that the blocked (1,1) triplet state is relaxed to the (0,2) triplet state (see Supplementary Note 3 and Supplementary Fig. 2 for additional examples of photo-electron blockade signals in the lower intermediate fields).

Figure 3b shows the main result on the probability of detecting a photo-electron blockade signal in *I*_sensor_ (blocked probability) for five different polarizations and two opposite magnetic fields. Each data point is obtained from ~50 photo-electron trapping signals out of ~1000 or more pulse irradiations. We set a threshold time of ~10 ms to distinguish the PSB signal from the signal of the anti-parallel spin transition of (1,1)-(0,2). The obtained PSB probabilities show a sinusoidal dependence when changing the photon polarization from the left-handed to the right-handed circular polarization, *σ*^−^ to *σ*^+^ via *H* linear polarization. Additionally, the probability curve shows an opposite behavior when the polarity of the magnetic field is reversed. This can happen because the initialized spin becomes opposite, while the photo-generated electron spin is not affected. The correspondence between polarization and spin indicates that a *σ*^−^ (*σ*^+^) photon creates an electron spin pointing in the positive (negative) direction, which is expected from the angular momentum transfer in the HH band excitation depicted in Fig. 1a.

## Discussion

Here we discuss the fidelity of the angular momentum transfer. We evaluate the fidelity of 63% by comparing the ideal detection against the worst case of measuring the opposite spin in Fig. 3b. The probabilities used for the calculation are averaged over the four combinations of circular polarization and field, e.g. *σ*^−^ and *σ*^+^ polarization and positive and negative fields for detecting the spin-blocked signal, and the same for detecting the un-blocked signal. The two most likely errors come from spin-relaxation during the measurement stage and thermalization in the (0,↑) initialization stage. From measurement of the tunneling time constants with no irradiation, we obtain *τ*_slow_ = 324 ± 1 ms (corresponding to the parallel spin *T*_1_) and *τ*_fast_ = 5.68 ± 0.02 ms, and calculate each fidelity of 93.0% (98.4%) for the parallel (anti-parallel) spin detection. Here we ignore the signal integration time. Asymmetry of the fidelity between the two spin configurations appears when we minimize the average error by adjusting the threshold time (see Supplementary Note 2 for derivations). Next for the thermal initialization, we prepare the (0,↑) state just by keeping an electron in the right dot, in a similar manner to previous work performed using an optical technique^24,25^. From the electron temperature of 100 mK (8.6 μeV) and Zeeman energy of 11.3 μeV in our experiment we evaluate the initialization fidelity of 79%. This error may be reduced by increasing the magnetic field of the in-plane direction but not the out-of-plane direction. We may further improve the fidelity, measurement time, and robustness of spin detection by utilizing this spin non-destructiveness^26,27^.

For the HH excitation in this condition, we think of the angular momentum transfer as a classical condition. The HH spin splitting is negligible due to zero *g*-factor, therefore if we irradiate a superposition of $|\sigma ^{-}> _{{\mathrm{ph}}}$ and $|\sigma ^{+}> _{{\mathrm{ph}}}$, this state has access to both spins of the HH and electrons^13^. The resulting electron spin will be entangled with the hole spin upon excitation, i.e. $\alpha |\sigma ^{-}> _{{\mathrm{ph}}} + \beta |\sigma ^ + > _{{\mathrm{ph}}} \rightarrow \alpha | \!+\! 1/2 > _{\mathrm{e}} \otimes | \!-\! 3/2 > _{{\mathrm{HH}}} + \beta | \!-\! 1/2 > _{\mathrm{e}} \otimes | \!+\! 3/2 > _{{\mathrm{HH}}}$. Then due to the short coherence time of the hole spin, the electron spin would also lose its phase information without interacting with its environment^28^. Meanwhile, the electron spin orientation may well be preserved because the HH escapes to the environment in a short time such that the spin exchange with the electron spin is small. Our observation in the PSB probability peaking at the circular polarization supports this claim of electron spin preservation. In the future, the transfer to spin orientation could be combined with down converted pair of photons, that can form a pair of single photon and a photo-electron pair^29^, for generation of classically correlated single spins. Another important path is to preserve the electron spin phase information after hole separation^28^. The requirement is to excite one of the spin-split LH band under an in-plane magnetic field to disentangle the hole spins while achieving symmetric excitation of the spin basis. After such feasible adjustment, the transfer can be applied for measuring entangled spin pairs and for efficient long-distant quantum communication using gate-defined QDs^13,30,31^.

In conclusion, we observed that the single electron spin created from a single circularly polarized photon has a correlation expected from the selection rules deduced from the quantum well structure of the host GaAs. Utilizing the two-electron spin blockade in the tunnel resonant condition served to project a single spin created by the photon and to justify the stability of the energy levels during detection. This progress may be integrated with the fast and remote optical investigations of single spins, which is promising as a classical and quantum nano-scale electronic platform.

## Methods

### Measurement sample

A DQD and a nearby QD charge sensor are fabricated in the QW. The QDs are defined by the surface Schottky gate electrodes shown in Fig. 1b. The current through the charge sensor, *I*_sensor_, detects the change in the charge configuration due to the event of photo-electron trapping in the DQD with a response time of 100 μs or less. Photon pulses with width of 3 ps are produced from a wavelength-tunable Ti:sapphire laser, and irradiated onto left dot through a 400-nm-diameter aperture in the metal mask. The device is placed in an optically accessible dilution refrigerator with base temperature of 25 mK. The QW wafer used here has a 7.3-nm-thick GaAs QW layer between a 95 nm-thick Al_0.34_Ga_0.66_As barrier layer above and a 100-nm-thick Al_0.34_Ga_0.66_As barrier layer beneath. The bottom thick barrier avoids excess photo-electrons coming from a thick GaAs buffer beneath the QW. An external magnetic field *B* is applied in the out-of-plane direction to the DQD device. The electron *g*-factor of the QW estimated for this field direction is |*g*_⊥_| = 0.12^32^ (see Supplementary Note 1 and Supplementary Fig. 1 for more details about the electronics and optics).

## Supplementary information

Supplementary Information

## Data Availability

The authors declare that the data supporting the findings of this study are available within the paper and its supplementary information files.
