# Supplementary material for: Angular momentum transfer from photon polarization to an electron spin in a gate-defined quantum dot
Source: Nat Commun. 2019 Jul 16;10:2991. doi: 10.1038/s41467-019-10939-x (PMC6635371; doi:10.1038/s41467-019-10939-x)
Supplement: Supplementary file 1 — Supplementary Information [file 41467_2019_10939_MOESM1_ESM.pdf]

Supplementary Information

Angular momentum transfer from photon polarization to an electron spin in a gate-defined quantum dot

Fujita et al.

Supplementary Note 1 Details on the sample and optical set-ups

We use a two-dimensional electron layer formed in a 7.3-nm-thick GaAs quantum well (QW), sandwiched by two AlGaAs barriers grown on a (001) GaAs substrate. The layer sequence from the surface is as follows: 5 nm GaAs capping layer, 65 nm Si doped $Al_{0.34}Ga_{0.66}As$ layer, 30 nm undoped $Al_{0.34}Ga_{0.66}As$ spacer, 7.3 nm GaAs well, undoped $Al_{0.34}Ga_{0.66}As$ barrier, and a thick GaAs buffer layer (same as in 'sample B' of Ref. 1). The Si doping is 1×10^{18} cm^{-3}, the carrier concentration is 2.1×10^{11} cm^{-2}, and the mobility is 0.1×10^{6} cm^{2}V^{-1}s^{-1}. The heavy-hole band is energetically well separated from the light hole band, relative to the incident photon bandwidth of 600 μeV. The heavy-hole excitation peak did not significantly change between a quantum well structure and a quantum dot (QD) formed on it, having a peak at 1.579 eV (wavelength 785 nm)[1]. The Zeeman energy used in the main measurements are well within the laser bandwidth, meaning that the spin-up and -down are still indistinguishable in terms of excitation energy.

Here, we define the laser beam propagation direction and external magnetic field (*B* field) axis pointing positive towards the backside of the sample (*z*-axis) and perpendicular to the plane (*x*-*y* plane) of the QD. The angular momentum of the electron spins and photon circular polarizations are also defined positive along this *z*-axis. All in units of $\hbar$, the heavy-hole states have angular momentum of ±3/2, from synthesizing the orbital angular momentum ±1 and the spin angular momentum ±1/2. The circularly polarized photon has angular momentum of ±1, which only interacts with the orbital motion of the electron. Photoexcitation fulfils the conservation of angular momentum when exciting an electron to the conduction band which has orbital angular momentum 0. Thus, we obtain the polarization to spin relation, as in Fig. 1a of the main text, where an angular momentum +1/2 electron spin is excited with a -1 angular momentum photon (σ^-) and an angular momentum -1/2 electron spin is excited with a +1 angular momentum photon (σ^+) [2]. For convenience, in the main text we used the term spin-up for an electron spin favouring the *B* field direction, and vice versa, which not necessarily corresponds to the sign of the angular momentum. For instance, in a negative *B* field spin-up has -1/2 angular momentum.

We tuned the optics so that a single photo-electron trapping signal is observed roughly every 30 shots on average, which was a suitable condition with low probability when aiming for single photon trapping. The photon flux calculated from the laser power used in this condition gave ~3 photons reaching the QD area per shot, which coincides with the expected quantum efficiency of the QW ~1 %. The Ti:sapphire laser provides trains of pulses and the pulse picker extract the desired number of pulses. We adjust the laser output power and the number of pulses to tune the final number of photons arriving at the sample. Additionally, two mechanical shutters are placed to sufficiently block the remaining photons. The shutter response ~30 μs is short enough so that in the end the total number of photons is fixed within the charge sensor resolution.

Using a lens mounted on the sample holder, the beam is focused to a diameter of 7 μm on the sample surface (Supplementary Figure 1a). The persistent photoconductivity largely reduced compared to Ref. 1 by focusing the laser beam smaller than the 30 × 30 μm^2 area (and 200 nm thick) Ti/Au metal mask. A fast steering mirror is placed below the cryostat to direct the laser on the QD. The polarization phase shift between horizontal and vertical polarization at the mirror is measured as 0.06 rad at 785 nm wavelength. This effect is negligible, less than 0.1 % reduction, for the sinusoidal amplitude of Fig. 3b in the main text.

We adjusted the inter-dot and side barrier tunnelling rates by observing the real-time traces to be able to follow the photo-electron dynamics in a non-equilibrium state. All tunnelling rates are kept slower than the measurement bandwidth 10 kHz to observe tunnelling events in real-time. Especially the rates for the source and drain barriers are low, on the order of hertz. The coupling to the left lead is made sufficiently weaker to keep the spin-blocked (1,1) state in the DQD, to observe a spin-flipped tunnelling to (0,2). The right barrier is set similarly to easily observe multiple tunnelling events between (1,1) and (0,2). The inter-dot coupling is tuned around a few 100 Hz to kHz depending on the experiment. This value sets the time scales of the spin dependent charge dynamics that we observe here, and the signal-to-noise ratio becomes better as we lower this rate. In principle, this tuning of inter-dot equally affects the tunnelling rates of both spins, therefore does not adequately differ the spin detection fidelity in terms of spin relaxation time. This fact was checked up to our observation from 100 μs to 10 ms range for the tunnelling times having zero spin[3–5]. We pause on the order of seconds between each single-shot measurement to make sure the excess electrons escape to the reservoir. This waiting time also ensures the relaxation of the single electron spin at the initialization stage in (0,1).

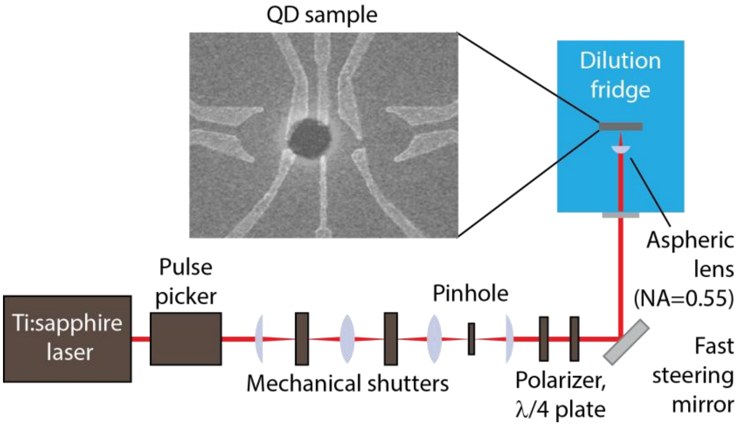

Supplementary Figure 1| Schematic of the optical set-up to irradiate polarized photons on the sample in a single-shot manner. We synchronize the pulse picker, mechanical shutters, and the voltage meter to obtain real-time signals. The laser transmits through a transparent window on the cryostat and focused with a lens placed close to the sample. The sample image shows the overlaid scanning electron micrograph image of the surface gate patterns and the metal mask.

Supplementary Note 2 Calculation of single-spin read-out fidelities

In this section we discuss the fidelities of the Pauli spin blockade (PSB) measurement used for single-spin detection and aim to calculate the optimal threshold time t_{th} which is needed to distinguish the spin states. The measurement of the PSB lifetime were performed at dark to obtain a large number of data points needed to create the double-exponential histogram (for the (1,1) residing times in Fig. 2b and 2c in the main text). This gives time constants, τ_{slow} and τ_{fast}, which we attribute to a parallel spin lifetime and an anti-parallel spin tunnelling time, respectively.

The detection fidelities are derived individually for the parallel and anti-parallel spin states, which also depend on the spin state to be measured. We start from the probability of finding a certain two-electron spin state, $A^*\tau_{slow}$ for parallel and $B^*\tau_{fast}$ for anti-parallel spin states. By setting a threshold time t_{th}, each of the error rate for detecting the parallel and anti-parallel spin states become,

$$A\int_0^{t_{\mathrm{th}}} e^{-t/\tau_{\mathrm{slow}}}\,dt \quad \text{and} \quad B\int_{t_{\mathrm{th}}}^{\infty} e^{-t/\tau_{\mathrm{fast}}}\,dt.$$

We optimize t_{th} under the condition that the spins have equal distribution ($A^*\tau_{slow}$ and $B^*\tau_{fast} = 1/2$), to equally account for the spin states created by the various photon polarization. By solving the minimization problem for the sum of the error rate, we yield,

$$t_{\mathrm{th}} = \frac{\tau_{\mathrm{slow}}\tau_{\mathrm{fast}}}{\tau_{\mathrm{slow}} - \tau_{\mathrm{fast}}} \ln\left(\frac{\tau_{\mathrm{slow}}}{\tau_{\mathrm{fast}}}\right).$$

Inserting this threshold value back the error rate formula gives,

$$A\tau_{\mathrm{slow}}\left(1-r^{\frac{1}{1-r}}\right),$$

for the parallel spin detection error rate, where $r=\frac{\tau_{\mathrm{slow}}}{\tau_{\mathrm{fast}}}$, and,

$$B\tau_{\mathrm{fast}}r^{\frac{r}{1-r}},$$

for the anti-parallel spin detection error rate.

The above formula gives the two-spin measurement error during the photo-electron-spin measurement. Applying this to the final tunnel rate conditions in the main text (τ_{fast} = 5.68 ± 0.02 ms and τ_{slow} = 324 ± 1 ms), we derive the parallel-spin detection fidelity 93.04 ± 0.04 % and similar for the anti-parallel spin 98.37 ± 0.02 % using an optimum threshold time t_{th} = 23.4 ms. As a consequence of the lower fidelity to detect parallel spins, the average probability can be lower than 0.5, as found in Fig. 3a of the main text. Another factor of error in the irradiation measurements can come from a fast escape of the excess electron to the reservoir, faster than the threshold time. We assume that this error is small owing to the slower rate than the spin dynamics.

Supplementary Note 3 Additional material on blockade signals of photo-electron spins

Photo-generated electrons should undergo a comparable spin-dependent tunnelling as to the electrons supplied from the reservoir. To verify this similarity, we detect the spin blockade of the photo-electrons and measure the blockade lifetime as we vary the magnetic field. Supplementary Figure 2a shows the selected traces of parallel-spin detection upon photo-electron trapping. We observe a relatively long residing time of the (1,1) state, which corresponds to spin blockade, in contrast to the repetitive inter-dot tunnelling dynamics of anti-parallel spins. Blockade time improved up to ~100 ms as we increased the external field up to 800 mT. This small dataset visualizes the increase of blockade time by the weakening of the effect from the hyperfine interaction[5].

The time constant ratio, τ_{slow} / τ_{fast}, important for the spin detection fidelity, no longer significantly increased above 400 mT. Supplementary Figure 2b shows the histograms of the (1,1) residing times after photo-electron trapping. The un-blocked signals are intentionally excluded from the dataset for clarity around 0 ms. Data for each field are individually plotted with different bin size in order to overlay the plots. We judge from these exponential histograms that the slopes are similar in log scale, agreeing to the fact that a single time constant represents the photo-electron blockade lifetimes due to spin-orbit interaction[3,4]. Overall, photo-electrons create spin states that qualitatively follow the spin physics of PSB studied for the thermally injected electron spins from the leads.

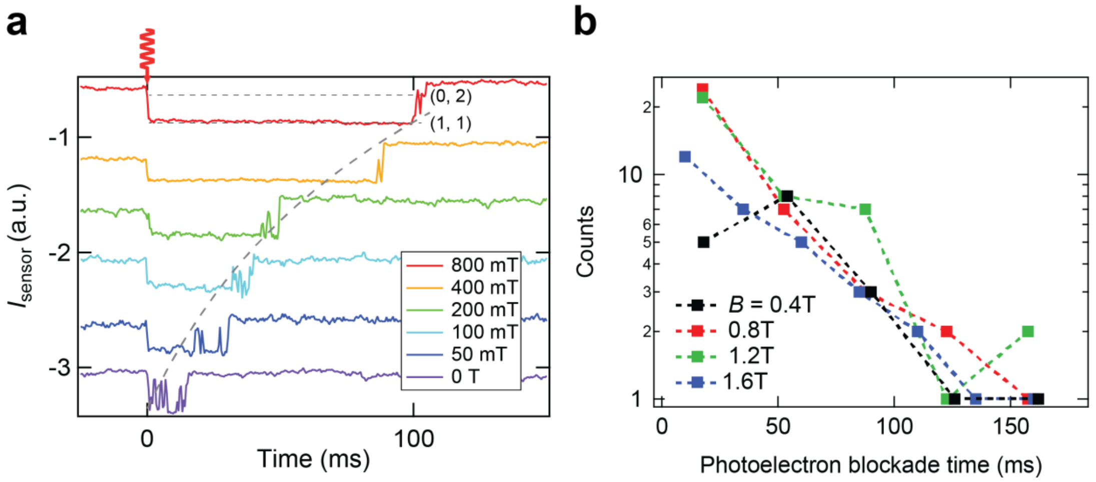

Supplementary Figure 2| a, Examples of single-shot photo-electron trapping signals that we intentionally picked to give an image of the photo-electron-spin blockade from zero to 800 mT. Around this range of low field, a monotonic increase in the blockade lifetime is expected due to the hyperfine interaction (dashed curve is a guide to the eye). The right barrier was lower than the left to allow faster initialization to (0,1) after relaxation to (0,2). **b**, Histograms of the (1,1) state residing time after photo-electron trapping at different fields from and above B = 400 mT. At these higher fields we expect a constant blockade life time due to the spin-orbit interaction. Data are shown for residing times above some threshold to clarify the blockade signals.

Supplementary Note 4 Additional discussion on the measurement fidelities

In the following, we discuss the fidelity of the result in Fig. 3b of the main text based on the different stages of the measurement, (Initialization) photon-polarization preparation, (Conversion) polarization-to-spin conversion at the QD interface, and (Detection) single photo-electron-spin read-out. The first suffers from polarization distortion errors, which occur at the room-temperature optics and/or at the mask aperture and gate electrodes on the semiconductor surface, and is estimated to be less than a percent based on our additional measurement and FDTD simulations.

The last detection stage is divided similarly to three stages but in the spin context, corresponding to the schematic in Fig. 1c of the main text: i) single-spin initialization, ii) photo-electron-spin loading to the QD after conversion, and iii) parallel and anti-parallel spin detection. According to the main text, the spin initialization fidelity is i) 79 %, and the parallel- (anti-parallel-)spin detection fidelity is iii) 93.0 % (98.4 %). Multiplying these two gives 77.7 % (73.5 %). To note, we had integrated the charge sensor signal for 100 μs to improve the signal-to-noise ratio at the specified tunnel coupling. This value broadens the shape of the double-

exponential histogram in the analysis, and possibly disturbs the optimized threshold time, set as 23 ms, but here the effect is considered negligibly small. Rather more crucial but difficult to quantify was the persistent photoconductivity effect due to the other photons in the pulse that were not trapped by the dot. When photons excite impurities, this more or less affects the dot potentials and hence also the inter-dot tunnel coupling. Relative to the many amount of impurity excitation, such effect may persist for an unpredictable time. We indeed observed by orders of hundreds of shots that the inter-dot energy detuning slightly shift and tunnel coupling increase, resolved in the telegraph signals present in the measurement. For now only manual feedback is taken, which would accompany additional measurement errors by a few to ten %.

The remaining stages of converting and loading the photo-electrons are somewhat fundamental to the heterostructure and the QD confinement. It seems that improvements are vital in this context to enable analysis of these values, such as by utilizing un-doped wafers suitable for spin qubit purposes[6] or increasing the photon-dot coupling efficiency by shaping the photons in an optical cavity[7].

Supplementary References

1. Morimoto, K. *et al.* Single photoelectron detection after selective excitation of electron heavy-hole and electron light-hole pairs in double quantum dots. *Phys. Rev. B* **90,** 085306-1–5 (2014).
2. Meier, F. & Zakharchenya, B. P. (ed). *Optical Orientation*. (Elsevier, Amsterdam, 1984).
3. Maisi, V. F. *et al.* Spin-Orbit Coupling at the Level of a Single Electron. *Phys. Rev. Lett.* **116,** 136803-1–5 (2016).
4. Fujita, T. *et al.* Signatures of Hyperfine, Spin-Orbit, and Decoherence Effects in a Pauli Spin Blockade. *Phys. Rev. Lett.* **117,** 206802-1–6 (2016).
5. Johnson, A. C. *et al.* Triplet-singlet spin relaxation via nuclei in a double quantum dot. *Nature* **435,** 925–928 (2005).
6. Mondal, S. *et al.* Field-effect-induced two-dimensional electron gas utilizing modulation-doped ohmic contacts. *Solid State Commun.* **197,** 20–24 (2014).
7. Chen, X., Nabet, B., Quaranta, F., Cola, A. & Currie, M. Resonant-cavity-enhanced heterostructure metal-semiconductor-metal photodetector. *Appl. Phys. Lett.* **80,** 3222–3224 (2002).
